# Supplementary material for: The Impact of Dietary Factors on the Sleep of Athletically Trained Populations: A Systematic Review
Source: Nutrients. 2022 Aug 10;14(16):3271. doi: 10.3390/nu14163271 (PMC9414564; doi:10.3390/nu14163271)
Supplement: Supplementary file 1 [file nutrients-14-03271-s001.zip › Quality Assessment- Table S2.pdf]

Supplementary Table S2. Quality Assessment

Author	Validity Questions										Quality Rating
	1	2	3	4	5	6	7	8	9	10	
Ormsbee et al. (2016)[76]	🟢	🟢	🟢	🔴	🟢	🟢	🔴	🔴	🟢	🟢	ø
Black et al. (2018)[75]	🟢	🟢	🔴	🟢	🟢	🟢	🟢	🔴	🟢	🟢	ø
Shamloo et al. (2019)[74]	🟢	🔴	🔴	🔴	🔴	🔴	🟢	🔴	🔴	🟢	-
Kasper et al. (2020)[77]	🟢	🟢	🟢	🟢	🟢	🟢	🔴	🟢	🟢	🔴	ø
Morehen et al. (2021)[70]	🟢	🟢	🟢	🔴	🔴	🟢	🟢	🟢	🟢	🟢	+
Harnett et al. (2021)[72]	🟢	🟢	🟢	🔴	🟢	🟢	🔴	🟢	🟢	🟢	ø
Quero et al. (2021)[73]	🟢	🟢	🟢	🟢	🟢	🟢	🟢	🟢	🟢	🟢	+
Wangdi et al. (2021)[71]	🟢	🟢	🟢	🔴	🟢	🟢	🔴	🔴	🔴	🟢	ø
MacInnis et al. (2020)[43]	🟢	🔴	🟢	🔴	🟢	🟢	🟢	🟢	🟢	🟢	ø
Oikawa et al. (2019)[60]	🟢	🟢	🟢	🟢	🟢	🟢	🟢	🟢	🟢	🟢	+
Miles et al. (2021)[61]	🟢	🔴	🟢	🟢	🟢	🟢	🟢	🟢	🟢	🟢	ø
Ferguson et al. (2022)[59]	🟢	🟢	🟢	🔴	🟢	🟢	🟢	🟢	🟢	🟢	+
Vandenbogaerde & Hopkins (2010)[67]	🟢	🟢	🟢	🔴	🟢	🟢	🔴	🔴	🟢	🟢	ø
Miller et al. (2014)[63]	🟢	🟢	🟢	🟢	🔴	🟢	🟢	🟢	🟢	🟢	+
Ali et al. (2015)[68]	🟢	🔴	🟢	🔴	🟢	🟢	🟢	🟢	🟢	🟢	ø
Dunican et al. (2018)[64]	🟢	🟢	🟢	🟢	🟢	🟢	🟢	🟢	🟢	🟢	+
Ramos-Campo et al. (2019)[65]	🟢	🟢	🟢	🔴	🟢	🟢	🟢	🟢	🟢	🟢	+
Raya-Gonzalez et al. (2021)[69]	🟢	🟢	🟢	🔴	🟢	🟢	🔴	🟢	🟢	🟢	ø
Caia et al. (2021)[66]	🟢	🟢	🟢	🔴	🟢	🟢	🟢	🟢	🟢	🟢	+
Hoshikawa et al. (2018)[81]	🟢	🔴	🟢	🟢	🟢	🟢	🟢	🟢	🟢	🟢	ø
Knufinke et al. (2018)[80]	🟢	🟢	🟢	🟢	🟢	🟢	🟢	🟢	🟢	🟢	+
Monma et al. (2018)[78]	🟢	🔴	🟢	🟢	🟢	🟢	🟢	🟢	🟢	🟢	ø
Silva & Paiva (2018)[62]	🟢	🟢	🟢	🟢	🟢	🟢	🟢	🟢	🟢	🟢	+
Yasuda et al. (2019)[83]	🟢	🟢	🟢	🟢	🟢	🟢	🔴	🟢	🟢	🟢	ø
Monma et al. (2021)[79]	🟢	🟢	🟢	🟢	🟢	🟢	🟢	🟢	🟢	🟢	+
Hoshino et al. (2022)[82]	🟢	🟢	🟢	🟢	🟢	🟢	🟢	🔴	🟢	🟢	+
Moss et al. (2022)[84]	🟢	🟢	🟢	🟢	🟢	🟢	🟢	🟢	🟢	🟢	+
Tinsley et al. (2019)[85]	🟢	🟢	🟢	🟢	🟢	🟢	🟢	🟢	🟢	🟢	+
Louis et al. (2016)[55]	🟢	🟢	🟢	🔴	🔴	🟢	🟢	🟢	🟢	🟢	+
Killer et al. (2017)[56]	🟢	🟢	🟢	🟢	🟢	🟢	🟢	🟢	🟢	🟢	+
Leyh et al. (2018)[58]	🟢	🟢	🟢	🟢	🟢	🟢	🟢	🟢	🟢	🟢	+
Vlahoyiannis et al. (2018)[57]	🟢	🟢	🟢	🔴	🟢	🟢	🟢	🟢	🟢	🟢	+
Daniel et al. (2019)[40]	🟢	🟢	🟢	🔴	🔴	🟢	🟢	🟢	🟢	🟢	+
Falkenberg et al. (2021)[37]	🟢	🟢	🟢	🔴	🟢	🟢	🟢	🟢	🟢	🟢	+
Condo et al. (2022)[54]	🟢	🟢	🟢	🔴	🟢	🟢	🟢	🟢	🟢	🟢	+

Quality rating symbols indicate a positive (+), neutral (ø), or negative (-) study rating.
